# Valorization of Animal-Derived By-Products Through Microencapsulation of Heme and Non-Heme Iron by Vacuum Foam Drying: Development of Functional Gummy Candies

**DOI:** 10.3390/molecules31132322

**Published:** 2026-07-02

**Authors:** Carlos A. Ligarda-Samanez, Eliana Villano-Limache, David Choque-Quispe, Elibet Moscoso Moscoso, Henry Palomino Rincón, Fredy Taipe Pardo, José C. Arévalo-Quijano, Dante Fermín Calderón Huamaní, Jackson M’coy Romero Plasencia, Justina Cervantes Carrión, Reynaldo Sucari-León, Jorge Apaza-Cruz, Daniela Isabel Dayan Ortega-Révolo

**Affiliations:** 1 Nutraceuticals and Biomaterials Research Group, Universidad Nacional José María Arguedas, Andahuaylas 03701, Peru; dchoque@unajma.edu.pe (D.C.-Q.); emoscoso@unajma.edu.pe (E.M.M.); hpalomino@unajma.edu.pe (H.P.R.); ftaipe@unajma.edu.pe (F.T.P.); jcarevalo@unajma.edu.pe (J.C.A.-Q.); dante.calderon@unica.edu.pe (D.F.C.H.); jackson.romero@unsch.edu.pe (J.M.R.P.); jcervantesc@utea.edu.pe (J.C.C.); rsucari@unah.edu.pe (R.S.-L.); jlapaza@unap.edu.pe (J.A.-C.); dortegar@continental.edu.pe (D.I.D.O.-R.); 2 Department of Education and Humanities, Universidad Nacional José María Arguedas, Andahuaylas 03701, Peru; 3 Department of Environmental Engineering, Universidad Nacional San Luis Gonzaga, Ica 11001, Peru; 4 Department of Mathematics and Physics, Universidad Nacional de San Cristóbal de Huamanga, Ayacucho 05000, Peru; 5 Faculty of Health Sciences, Universidad Tecnológica de los Andes, Abancay 03001, Peru; 6 Engineering and Management Faculty, Universidad Nacional Autónoma de Huanta, Ayacucho 05000, Peru; 7 Academic Department of Electronic Engineering, Universidad Nacional del Altiplano, Puno 21001, Peru; 8 Faculty of Management Sciences, Universidad Continental, Huancayo 12000, Peru

**Keywords:** food fortification, iron-deficiency anemia, gelled matrices, structural stability, multicomponent systems, encapsulation technology

## Abstract

Iron deficiency and associated anemia remain major public health concerns, requiring innovative food fortification systems with adequate technological and sensory performance. This study aimed to develop a multicomponent fortified gummy candy using heme iron from *Cavia porcellus* erythrocytes, non-heme iron from Feranin^®^, and elderberry juice, integrated through microencapsulation and stabilized by vacuum foam drying. Erythrocytes were isolated, dehydrated, and microencapsulated in a tara gum–maltodextrin matrix, yielding a powder with 1.49 mg Fe/g dry matter. The microencapsulates exhibited compact morphology, lower polydispersity, and negative ζ potential, indicating suitable surface stability. Three gummy formulations (F1–F3) were prepared with different proportions of encapsulated erythrocytes, non-heme iron, and elderberry juice. Iron content increased significantly from 0.21 to 0.89 mg Fe/g of gummy candy. The formulations showed variations in water activity (0.84–0.88), moisture (33.79–40.22%), pH (4.64–6.15), soluble solids (41.00–46.67 °Brix), phenolic compounds (0.80–1.14 mg GAE/g), flavonoids (0.13–0.27 mg QE/g), and antioxidant capacity (1.53–3.75 µmol TE/g). FTIR and SEM confirmed structural preservation and matrix integration. Sensory evaluation showed comparable overall acceptability among formulations, with F3 showing higher mean ranks for flavor and texture. Overall, vacuum foam drying was a feasible strategy for valorizing animal-derived by-products in fortified gelled confectionery.

## 1. Introduction

Iron has been identified as one of the most difficult micronutrients to successfully incorporate into processed foods, because its various compounds exhibit significant variations in behavior within the food matrix and can cause undesirable changes in color and flavor, which represents a major technological barrier in the design of effective fortified foods [1]. These limitations are associated with the susceptibility of food systems to oxidation processes during processing and storage, in which reactive oxygen species can induce structural modifications in proteins and other matrix components, promoting phenomena such as aggregation, fragmentation, or the formation of derivatives that alter their physicochemical properties and functional stability [2]. In this context, the interaction between iron and dietary proteins depends on the iron-to-protein ratio, which influences both the binding capacity and the valence state of the metal in the complex; it has been observed that increases in iron content can induce protein aggregation and alter the structural and thermodynamic stability of the system [3]. Likewise, the stability and bioavailability of bioactive compounds in food systems depend largely on their molecular composition and their interaction with the various components of the dietary matrix, which can exert additive, synergistic, or antagonistic effects that modulate their functional behavior and bioavailability [4].

From a formulation engineering perspective, functional foods can be designed as multicomponent systems in which the final functionality depends on interactions among their constituents rather than solely on the isolated addition of a nutrient. Evidence indicates that microencapsulation technologies protect active compounds and control their release, facilitating their handling, stability, and performance in complex food matrices [5]. In the case of iron, these strategies are particularly relevant because encapsulation can act as a barrier against interactions with matrix components and improve its soluble fraction during digestion, especially when combined with chemical modulators such as vitamin C [6,7,8]. Likewise, comparative studies have shown that iron bioavailability depends on its chemical form, with heme iron having a greater impact on certain indicators of iron status than non-heme iron, highlighting the importance of designing systems that strategically integrate different mineral sources [9,10]. Accordingly, the combination of heme and non-heme iron enables the strategic use of their complementary characteristics, integrating the higher absorption efficiency of the former with the versatility of the latter, whose performance may be enhanced by compounds such as vitamin C from elderberry juice. In addition, blood from *Cavia porcellus* is used as an alternative source of heme iron, obtained from authorized facilities under regulated sanitary conditions.

Encapsulation therefore represents a relevant technological strategy aimed at protecting active compounds, improving their stability during processing and storage, modulating their release, and enhancing their compatibility with complex food matrices, especially when incorporating chemically reactive or sensorially challenging ingredients [11,12]. However, several conventional drying methods used to obtain encapsulated ingredients present significant limitations, including exposure to high temperatures, longer processing times, and high operating costs, which can compromise the structural and functional integrity of encapsulated bioactive compounds [12,13]. In response to this, vacuum foam drying, particularly its integration with vacuum conditions, emerges as an alternative for intensifying drying processes, as it transforms liquid or semi-liquid systems into aerated and highly porous structures that increase interfacial area, accelerate mass transfer, promote moisture diffusion, and enable drying at more moderate temperatures, thereby reducing thermal degradation and better preserving the quality of the final product [13,14,15,16]. Previous studies have shown that vacuum foam drying can produce high-quality powders by rapidly and gently removing water. In contrast, kinetic analyses of vacuum foam drying systems confirm that temperature and layer thickness decisively control the drying rate, effective diffusivity, and retention of physicochemical properties [15,16].

Despite the growing number of studies on iron fortification and the use of encapsulation technologies in food systems, most research has focused on individual sources of iron or on simplified model systems designed primarily to improve iron stability and/or bioaccessibility during digestion [17,18]. Microencapsulation has been extensively studied as a technological strategy to protect iron compounds, reduce undesirable sensory effects, and facilitate their incorporation into foods; however, most reported studies use non-heme iron salts or relatively simple model matrices rather than more complex multicomponent formulations [11,17]. Similarly, various drying technologies have been evaluated for the stabilization of encapsulated bioactive compounds; however, applications of foam drying have focused primarily on plant extracts, fruit juices, or fruit pulps, with little exploration in mineral fortification systems [13,14]. Furthermore, although previous studies have independently analyzed ingredients rich in heme iron, the use of ascorbic acid as an absorption modulator, or the use of encapsulated systems for controlled nutrient release, the simultaneous integration of heme and non-heme iron sources combined with redox modulators within encapsulated structures and incorporated into gelled matrices has received very little attention in the scientific literature [13,17,19]. Consequently, the available knowledge remains fragmented and focused primarily on isolated technological components, rather than on the integrated design of functional systems. The fragmentation of current knowledge highlights the need for research focused on developing multicomponent systems in which formulation, encapsulation, and drying processes are jointly designed to control physicochemical interactions, enhance structural stability, and improve functional performance within the final food matrix.

Based on this gap, we hypothesized that integrating heme iron derived from *Cavia porcellus* blood erythrocytes, non-heme iron, and elderberry juice into a microencapsulated system stabilized by vacuum foam drying would improve iron compatibility with a gelled confectionery matrix, while preserving structural integrity and sensory acceptability. Therefore, the present study aimed to design, develop, and evaluate this multicomponent fortification system for incorporation into gummy-type gelled matrices. From a food engineering perspective, this work was approached as a proof-of-concept to assess the physicochemical, structural, bioactive, and sensory properties of the developed system under controlled conditions.

## 2. Materials and Methods

### 2.1. Materials and Reagents

The iron sources and natural raw materials used in this study were selected to formulate fortified gummy candies. Guinea pig (*Cavia porcellus*) blood, used as a source of heme iron, was collected under appropriate hygienic and sanitary conditions and provided by the Municipal Slaughterhouse of the district of San Jerónimo, province of Andahuaylas, Apurímac region (Peru), an establishment authorized by the National Agricultural Health Service (SENASA). In the Peruvian and Andean context, *Cavia porcellus* is a traditional food animal raised in family and commercial production systems for human consumption. Therefore, the use of its blood in this study was considered within a local food-production framework, and the material was obtained only from an authorized slaughterhouse under regulated hygienic and sanitary conditions.

Feranin^®^ (Quilab, Lima, Peru), a commercial supplement formulated as an oral syrup, was used as a source of non-heme iron. According to the manufacturer’s information, this product contains 50 mg of iron per 5 mL, supplied as ferric iron (Fe^3+^) in the form of 29% polymaltose ferric hydroxide (172.41 mg), in addition to sorbitol (0.28 g/mL) as an excipient. In this study, Feranin^®^ was selected as the source of non-heme iron due to its widespread use and stability in food formulations. It should be noted that, in preliminary trials, the use of ferrous sulfate was evaluated; however, it produced a pronounced metallic taste, so its use in the final formulations was ruled out.

Likewise, elderberries were harvested at consumption maturity in the district of Talavera, province of Andahuaylas, Apurímac region (Peru). They are used as a natural source of bioactive compounds and vitamin C in the formulation. All reagents used in the analyses were of analytical grade, while the food ingredients used in the formulation were suitable for food application.

The use of animal-derived material in this research was evaluated and approved by the Ethics Committee of the José María Arguedas National University through the Faculty of Engineering, via Resolution No. 486-2025-D-FI-UNAJMA, issued on 11 August 2025.

### 2.2. Microencapsulation by Vacuum Foam Drying

Guinea pig blood was collected and treated with sodium citrate (3 g/L) to prevent clotting. The sample was then filtered to remove solid residues and centrifuged at 10,000 rpm for 15 min to separate the cellular fractions. The resulting red blood cell pellet was washed with a 0.5% (*w*/*v*) NaCl solution to remove residual plasma and other impurities. Subsequently, the sediment was placed in Petri dishes and dried in a Binder VD56 vacuum oven (Tuttlingen, Germany) at 70 °C for 8 h. The resulting dry material was milled and sieved to obtain a homogeneous powder.

To prepare the encapsulating matrix, tara gum and maltodextrin were weighed and mixed at a 5:95 (*w*/*w*) ratio, then homogenized using an Ultra-Turrax homogenizer (Daihan, Wonju, Republic of Korea) until a uniform mixture was obtained. Then, 10 g of this matrix was dissolved in 100 mL of ultrapure water under continuous magnetic stirring, and 20 g of dried erythrocytes were incorporated, yielding a uniform suspension.

The suspension was stirred at 460 rpm for 10 min using a mixer until a stable foam formed. The foam formation was monitored to ensure its homogeneity and stability prior to drying, as these factors are critical for achieving consistent drying performance and structural integrity of the encapsulated material. The generated foam was evenly distributed into silicone molds and dried in a vacuum oven at 70 °C under controlled vacuum conditions (reduced pressure relative to atmospheric pressure) for 5 h. Finally, the dried material was milled again to obtain a fine powder and stored in airtight plastic bottles at 18 °C until further use.

### 2.3. Preparation of Gummy Candies

The fortified gummy candies were prepared according to the formulations described in Table 1, which differ in their content of encapsulated red blood cells, non-heme iron, and elderberry juice, while the remaining ingredients of the gelled matrix were kept constant. Elderberry juice was obtained from previously selected fruits, which were sanitized with sodium hypochlorite solution (50 ppm for 5 min), blended using a food blender, and strained to remove skins and seeds. The juice was measured at 75 mL per formulation and used as the liquid component of the matrix. Feranin^®^ was added to each formulation in the proportions defined for each treatment.

In parallel, the gelling phase was prepared by hydrating neutral gelatin in water, then adding sucrose, glucose, isomalt, and glycerin, using a hot plate with a magnetic stirrer to ensure homogeneous dissolution. Sodium benzoate was added to this mixture as a preservative agent. The gelling phase and the elderberry juice were combined and pasteurized at 75 °C for 5 min in a water bath. Subsequently, the encapsulated erythrocytes were incorporated in the proportions corresponding to each formulation, followed by homogeneous mixing. Finally, the resulting mixture was poured into silicone molds (1 mL per unit), cooled under refrigerated conditions at 8 °C for 24 h, demolded, and stored under refrigeration until subsequent physicochemical and sensory analysis.

It should be noted that no control formulation was included in the present study, as preliminary trials with conventional iron sources, such as ferrous sulfate, produced a metallic taste that compromised the sensory acceptability of the gummy candies. Consequently, an iterative approach based on exploratory trials was adopted to obtain formulations with acceptable sensory characteristics. In addition, it was ensured that the formulations were technically feasible and that the proportions of the components allowed achieving iron contents potentially adequate to meet nutritional requirements. Due to this exploratory approach and the need to prioritize the product’s technological and sensory feasibility, a conventional mixture design was not used. Thus, three formulations (F1, F2, and F3) were selected as representative of the evaluated system. Although control formulations without iron or without encapsulation were not included, the study focused on the development and evaluation of the final product; therefore, the results should be interpreted within this scope.

### 2.4. Physicochemical Determinations

#### 2.4.1. Iron Content

A total of 200 mg of sample was taken and subjected to a digestion process using a mixture of hydrochloric acid (Spectrum Chemical Mfg. Corp., Bathurst, NB, Canada) and nitric acid (Spectrum Chemical Mfg. Corp., Bathurst, NB, Canada) in a microwave digester (SCP Science, Miniwave, QC, Canada). Subsequently, iron quantification was performed by inductively coupled plasma optical emission spectrometry (ICP-OES 9820 138, Shimadzu, Kyoto, Japan), using an external calibration curve ranging from 0 to 50 mg/L and an analytical wavelength of 239.56 nm [20].

#### 2.4.2. Water Activity (Aw)

It was determined using a HygroPalm23-AW meter from Rotronic (Bassersdorf, Switzerland). The equipment was previously calibrated with standard solutions following the manufacturer’s recommendations. Representative portions of each gummy candy sample were placed in the measurement chamber, where the equilibrium relative humidity was recorded, which the equipment itself automatically converts into Aw values through its internal calibration curves [21].

#### 2.4.3. Hydrogen Potential (pH)

For pH determination, 10 g of each gummy candy sample was homogenized in 50 mL of distilled water preheated to 45 °C. The pH of the mixture was measured with a calibrated potentiometer (Lab 885, SI Analytics, Mainz, Germany) [20].

#### 2.4.4. Soluble Solids

The soluble solids content was determined by refractometry. For this purpose, a portion of the sample was placed on the prism of an ABBE refractometer (Isolab, Wertheim, Germany), and the corresponding readings were recorded [20].

#### 2.4.5. Moisture

Moisture content was determined by the oven-drying method following AOAC procedure 950.10 [22]. Approximately 3.0 g of each gummy candy sample was placed in previously tared capsules and dried in an oven at 105 °C until constant weight was reached. Moisture was calculated based on the mass loss recorded during the drying process.

#### 2.4.6. Instrumental Color

The color of the gummy candy samples was evaluated using a benchtop colorimeter CR-5 (Konica Minolta, Tokyo, Japan), equipped with a reflectance accessory and a 40.5 × 60.0 mm measurement cell. Color parameters were recorded in the CIELAB color space as L* lightness, a* green–red coordinate, and b* blue–yellow coordinate [23].

#### 2.4.7. Total Phenolic Compounds

Total phenolic compounds were quantified using the Folin–Ciocalteu assay [24] employing gallic acid as the calibration standard. For the analysis, 900 µL of the sample extract was transferred into a test tube, followed by the addition of 2400 µL of ultrapure water, corresponding to a dilution factor of 3.666, 300 µL of 0.25 N Folin–Ciocalteu reagent, and 150 µL of 20% Na_2_CO_3_. The reaction mixture was allowed to stand for 15 min at room temperature in the absence of light. A blank solution was prepared in parallel using the same procedure, but replacing the sample with ultrapure water. Absorbance was measured at 755 nm using a UV–Vis spectrophotometer (Genesys 150, Thermo Fisher Scientific, Madison, WI, USA) [25].

(1) $$TPC=\frac{X\times V\times DF\times 100}{m\times DM}$$

where TPC represents the total phenolic content, X is the gallic acid-equivalent concentration obtained from the calibration curve, V is the extract volume, DF is the dilution factor, m is the sample mass, and DM is the dry matter content.

#### 2.4.8. Total Flavonoids

A quercetin standard solution prepared in ethanol was used to construct the calibration curve. For flavonoid extraction, 0.5 g of each gummy candy sample was mixed with 20 mL of 80% methanol and extracted at room temperature for 24 h under light-protected conditions. For quantification, 90 µL of the extract was mixed with 100 µL of AlCl_3_ solution and 4.81 mL of 80% methanol. Absorbance was measured at 425 nm using a UV–Vis spectrophotometer (Genesys 150, Thermo Fisher Scientific, Madison, WI, USA) [26].

#### 2.4.9. Antioxidant Capacity

Antioxidant capacity was determined using the DPPH assay (2,2-diphenyl-1-picrylhydrazyl stable radical), employing Trolox (6-hydroxy-2,5,7,8-tetramethylchroman-2-carboxylic acid) as the calibration standard. Prior to analysis, the spectrophotometer was calibrated with methanol, and the DPPH solution was adjusted to an absorbance of 1.1 ± 0.02 at 515 nm. Subsequently, 150 µL of the methanolic sample extract was mixed with 2850 µL of the adjusted DPPH solution and allowed to react for 15 min at room temperature in the absence of light. A blank was prepared using 150 µL of 80% methanol instead of the sample, combined with 2850 µL of the adjusted DPPH solution. Absorbance was measured at 515 nm using a UV–Vis spectrophotometer (Genesys 150, Thermo Fisher Scientific, Madison, WI, USA) [25].

(2) $$AC=\frac{X\times V\times 100}{m\times DM}$$

where AC is the antioxidant capacity, X is the antioxidant-equivalent concentration obtained from the calibration curve, V is the extract volume, m is the sample mass, and DM is the dry matter content.

### 2.5. Structural and Instrumental Characterization

#### 2.5.1. Scanning Electron Microscopy (SEM) and Energy Dispersive X-Ray Spectroscopy (EDS)

Morphology was examined by scanning electron microscopy using a Prism E equipment (Thermo Fisher Scientific, Waltham, MA, USA). Observations were carried out at an accelerating voltage of 25 kV and a magnification of 500×. The equipment was coupled to an energy-dispersive X-ray spectroscopy system, which allowed determination of the elemental composition of the analyzed particles’ surfaces [27].

#### 2.5.2. Particle Size and Polydispersity Index

The particle size of the samples was determined by laser diffraction using a Mastersizer 3000 analyzer (Malvern Instruments, Worcestershire, UK). For the analysis, 0.02 g of the sample was dispersed in 50 mL of isopropyl alcohol, and the suspension was stirred at 3000 rpm for 2 min. Subsequently, the mixture was subjected to ultrasonic treatment in a DU-220S bath (ARGO LAB, Milan, Italy) for 10 min at 40 kHz to promote particle dispersion. Measurements were carried out at 600 nm.

The polydispersity index (PDI) was calculated from the percentiles of the cumulative particle size distribution according to the following relationship:

(3) $$PDI=\frac{\left(D_{90}-\;D_{10}\right)}{D_{50}}$$

where $D_{10}$, $D_{50},$ and $D_{90}$ are the diameters corresponding to 10%, 50% and 90% of the cumulative particle size distribution, respectively [23].

#### 2.5.3. Zeta Potential (ζ)

This was determined by dynamic light scattering using a Zetasizer ZU3100 analyzer (Malvern Instruments, Worcestershire, UK). For the analysis, 0.02 g of the sample was dispersed in the corresponding medium, and the suspension was subjected to ultrasound for 10 min at 40 kHz to promote adequate particle dispersion. Measurements were carried out using a DTS1070 cell under the equipment’s standard operating conditions [23].

#### 2.5.4. Fourier-Transform Infrared Spectrophotometry (FTIR)

The functional groups present in the samples were analyzed by FTIR using a Nicolet IS50 spectrophotometer (Thermo Fisher Scientific, Waltham, MA, USA). Spectra were recorded in the mid-infrared range (4000–400 cm^−1^) with a resolution of 8 cm^−1^ and 32 scans per sample [20,27].

### 2.6. Affective Sensory Evaluation

Gummy candy samples were conditioned for 2 weeks to promote product stabilization prior to sensory analysis. Subsequently, acceptability was evaluated using a panel of 70 untrained consumers, selected by convenience sampling and with a balanced gender distribution (50% female and 50% male).

Samples were presented in portions of approximately 20 g on disposable plates and identified with random three-digit codes to avoid bias during evaluation. Each participant was provided with mineral water for mouth rinsing between samples. The tests were conducted in a well-lit environment, with adequate ventilation and free of odors that could interfere with the evaluation.

Acceptability was determined using a structured five-point hedonic scale: 1 = dislike very much, 2 = dislike, 3 = neither like nor dislike, 4 = like, and 5 = like very much. The evaluated attributes were overall appearance, flavor, texture, and overall acceptability. Sensory data were analyzed using nonparametric tests because the hedonic scale generated ordinal data. Differences among formulations were evaluated using the Friedman test, followed by pairwise comparisons with the Wilcoxon signed-rank test when significant differences were detected. Statistical significance was set at *p* < 0.05.

The minimum sample size was calculated a priori using Cochran’s formula for proportions, considering a 95% confidence level and a 10% margin of error. This calculation supported the number of participants included in the sensory evaluation. Before the evaluation, the procedure was explained to the participants, who signed the corresponding informed consent form to ensure their voluntary participation. The study was conducted under minimal-risk conditions [20,23].

### 2.7. Statistical Analysis

Data on physicochemical properties were analyzed using analysis of variance (ANOVA), followed by Tukey’s multiple-comparison test, with a significance level of 5%. All experiments were performed in triplicate to ensure the reliability and consistency of the results. Sensory evaluation data were analyzed using the Friedman test, followed by Wilcoxon signed-rank tests for pairwise comparisons when significant differences were detected. Statistical analyses were carried out using OriginPro 2026 software (OriginLab Corporation, Northampton, MA, USA) [20,23].

## 3. Results and Discussion

### 3.1. Characterization of Erythrocytes and Microencapsulates

The physicochemical and instrumental properties were evaluated to characterize the base ingredients used in the formulation of gummy candies. The total iron content determined in the erythrocytes was 4.00 mg Fe/g dry matter, higher than that reported in spray-dried guinea pig blood erythrocytes (3.30 mg Fe/g dry matter) [27] and also higher than that recorded in commercial bovine erythrocytes (2.49 mg Fe/g dry matter) [28]. These differences can be attributed to variations in the animals’ diet and physiological state, as well as to processing conditions and the purity of the erythrocyte fraction obtained.

In the microencapsulated system, a value of 1.49 mg Fe/g dry matter was obtained, which falls within the range reported for similar microencapsulates obtained by spray drying (1.35–3.56 mg Fe/g dry matter). This behavior can be attributed to the incorporation of the encapsulating materials into the solid matrix, leading to a relative dilution of iron in the final system [27,29]. Furthermore, the foam-drying method used in this study favored the concentration of solids in the microencapsulated matrix, thereby enhancing the retention of iron associated with hemoglobin.

**Figure 1:**
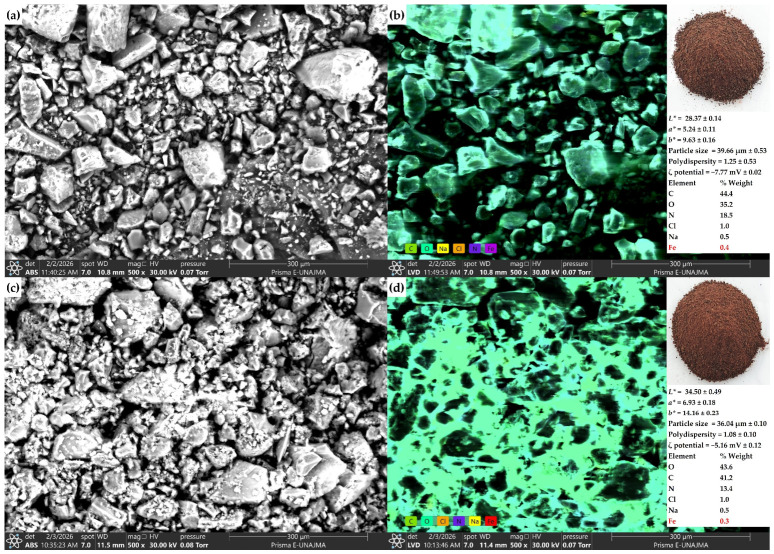
SEM micrographs of erythrocytes (**a**); SEM–EDS analysis and physicochemical and instrumental characterization of erythrocytes (**b**); SEM micrographs of microencapsulated erythrocytes (**c**); SEM–EDS analysis and physicochemical and instrumental characterization of microencapsulated erythrocytes (**d**).

Figure 1 summarizes the main physicochemical and instrumental parameters evaluated, including surface morphology (Figure 1a,c), color coordinates, particle size, polydispersity index, ζ-potential, and elemental composition (Figure 1b,d).

SEM micrographs showed that the erythrocytes exhibit particles with irregular morphology, angular edges, and heterogeneous surfaces (Figure 1a), characteristics of solid materials without added structuring agents [20]. In contrast, the microencapsulates exhibited a more compact and continuous structure, with partially coated particles and more integrated surfaces (Figure 1c), consistent with the formation of a continuous encapsulating matrix during vacuum foam drying [20,30].

The erythrocytes showed color parameters typical of a dark material, with a predominance of positive a* and b* coordinates, associated with reddish and yellowish tones and the presence of heme compounds [22,27]. In the microencapsulated system, higher lightness values were observed, along with increases in the chromatic parameters a* and b* (Figure 1d), indicating a visually lighter material with greater chromatic intensity. This behavior can be attributed to the incorporation of the encapsulating agents and to the surface redistribution of components during the vacuum foam-drying process [14,20].

The mean particle size of the erythrocytes was larger than that of the microencapsulated samples (Figure 1d). This slight reduction may be associated with the encapsulation process and vacuum foam drying, which favor particle fragmentation and matrix reorganization, promoting the formation of more homogeneous structures characteristic of dehydrated foamed matrices [13,14].

The erythrocytes exhibited a higher polydispersity index (Figure 1b), reflecting a broad distribution of particle sizes, consistent with the irregular nature of the original material [20]. In contrast, the microencapsulated system exhibited a lower index (Figure 1d), indicating a more uniform distribution attributable to the structuring effect of the encapsulating materials and the control of particle size during vacuum foam drying [13,14]. The ζ potential of the erythrocytes and the microencapsulated system showed negative values (Figure 1b,d), suggesting the presence of net surface charges that contribute to the system’s stability. Following encapsulation, a slight decrease in the magnitude of the ζ potential was observed, likely due to partial coating of the charged surfaces by the encapsulating materials [20,22,27].

Elemental analysis revealed that both systems were composed primarily of carbon (C), oxygen (O), and nitrogen (N), with the additional presence of sodium (Na) and chlorine (Cl), attributed to the sodium chloride in the saline solution used during erythrocyte extraction, as well as iron (Fe) associated with the heme group of hemoglobin present in guinea pig blood (Figure 1b,d). A slightly higher iron signal was detected in the erythrocytes compared to the microencapsulated system, consistent with the total iron content values obtained by ICP-OES. On the other hand, in the microencapsulates, the elemental distribution reflects the contribution of the encapsulating materials to the overall composition of the system [20,27].

### 3.2. Physicochemical Characterization of the Gummy Candies

The physicochemical properties of the formulated gummy candies are presented in Table 2. The iron content increased progressively across the formulations, from 0.21 mg Fe/g in F1 to 0.89 mg Fe/g in F3, consistent with the increase in the proportion of encapsulated erythrocytes and Feranin^®^ incorporated into the gelled matrix. However, these values were lower than those reported for gummy candies fortified with microencapsulated guinea pig blood erythrocytes in tara gum and maltodextrin via vacuum drying (1.96–2.63 mg Fe/g) [20]. This difference can be attributed to the lower erythrocyte proportion and the dilution effect of the multicomponent matrix, both of which influence the stability and bioavailability of iron in fortified foods [6,31].

Although iron content was quantified in this study, its bioavailability was not directly assessed. However, several studies have shown that microencapsulation can enhance iron stability and reduce undesirable interactions within the food matrix, thereby improving its bioaccessibility during digestion [17,32]. In addition, the presence of compounds such as vitamin C from elderberry juice may enhance the bioaccessibility of non-heme iron by reducing Fe^3+^ to more absorbable forms [33,34]. Therefore, the results should be interpreted within this scope, and further in vitro or in vivo studies are required to confirm these effects.

Water activity and moisture content decreased progressively from F1 to F3, although they remained within the high ranges typical of gelled confectionery due to the matrix composition. The moisture content exceeded the recommended range (15–30%), which may affect texture and increase susceptibility to microbial spoilage [35,36]. Reducing moisture improves firmness, decreases stickiness, and promotes product stability by limiting the water activity available for microbial growth [35,37,38]. In this regard, the consistency and stability of the foam during vacuum foam drying are key to the efficiency of the encapsulation process and the development of the final textural properties of the product. A more homogeneous foam structure may promote more uniform drying and contribute to the structural integrity of the encapsulated material, as reported in previous studies [39,40,41]. However, specific drying kinetics and rheological properties of the foam were not evaluated in the present study, which constitutes a direction for future research.

The pH of the gelled matrices showed a progressive increase from moderately acidic values in F1 to values close to neutrality in F3, which demonstrates the influence of the formulation’s composition on the system’s acid–base balance. This behavior may be associated with the relative decrease in elderberry juice, a source of organic acids, and the increase in components with lower acidity, which shifts the system toward higher pH values; furthermore, the buffering capacity of proteins and polysaccharides present in the matrix contributes to stabilizing the acid–base balance of the food system [2,4].

The soluble solids content decreased from F1 to F3 as the proportion of matrix components changed, reflecting a lower fraction of soluble compounds in the system. This behavior can be attributed to variations in the formulation, which influence the physicochemical properties of confectionery products and the concentration of dissolved solids [4,42].

Color measurements showed low and comparable L* values across the formulations, consistent with dark-colored gelled matrices. In contrast, the chromatic coordinates a* and b* showed moderate variations, which may be associated with differences in formulation composition [20,23].

Finally, phenolic compounds, flavonoids, and antioxidant capacity were measured; F1 and F2 showed comparable results for phenolic compounds and flavonoids, whereas F3 exhibited lower values. Regarding antioxidant capacity, the three formulations differed. These variations are associated with the elderberry juice content, which was highest in F1, followed by F2 and F3; this pattern is attributed to the fact that elderberry juice provides phenolic compounds and flavonoids; therefore, higher inclusion levels are expected to increase their concentration in the formulations. Likewise, antioxidant capacity follows this same trend, as it depends on these compounds [43,44]. Previous studies have reported that matrices rich in phenolic compounds, particularly fruit-derived matrices, show a close relationship between phenolic concentration and antioxidant capacity, as well as possible synergistic interactions among bioactive compounds that enhance their functional activity [45,46,47,48,49].

The incorporation of heme iron derived from *Cavia porcellus* blood into gelled matrices raises relevant considerations in terms of consumer acceptance and regulatory frameworks, particularly in direct consumption contexts. In this regard, the obtained results should be interpreted considering the experimental scope of the developed system. Future studies should further explore these aspects to assess its applicability in real-world scenarios. In this context, the absence of control formulations implies that the results should be interpreted in comparative terms among the evaluated formulations, rather than as effects independently attributable to encapsulation or the iron source. This approach reflects the exploratory nature of the study and the aim of assessing the performance of the multicomponent system under realistic formulation conditions.

Although the total iron content increased significantly in the formulations, this result should not be interpreted as direct evidence of bioavailability. Iron absorption depends on its chemical form, the food matrix, and the presence of compounds that may enhance or limit its utilization [1,50]. In this context, the combination of heme and non-heme iron, together with reducing compounds present in elderberry juice, could favor mineral availability; however, this hypothesis needs to be confirmed through specific assays [8,9]. Therefore, the results of the present study should be understood as evidence of successful iron incorporation and technological compatibility within a gelled matrix. Future studies should evaluate bioaccessibility and bioavailability through simulated gastrointestinal digestion, cellular models, or in vivo studies [51].

### 3.3. FTIR Analysis of Erythrocytes, Microencapsulates, and Gummy Candies

**Figure 2:**
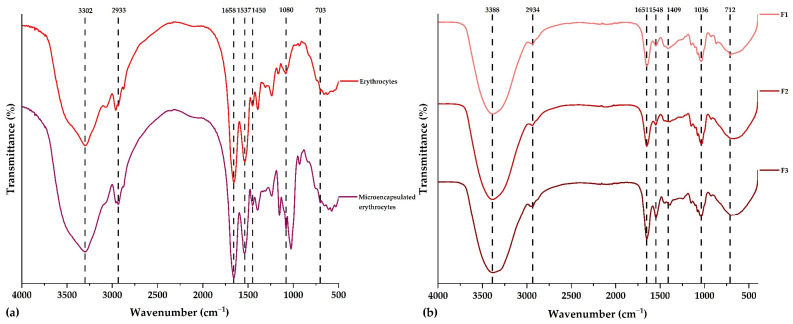
FTIR spectra of erythrocytes and microencapsulated erythrocytes (**a**), and gummy candies (**b**).

Figure 2a shows the FTIR spectra of non-encapsulated *Cavia porcellus* blood erythrocytes and erythrocytes microencapsulated within a tara gum–maltodextrin matrix. In both cases, a broad band is observed around 3302 cm^−1^, associated with O–H groups and, to a lesser extent, N–H groups, present in proteins and polysaccharides. The signals near 2933 cm^−1^ correspond to C–H groups of organic compounds, such as proteins, lipids, and carbohydrates. In the mid-infrared region, bands appear around 1658 and 1537 cm^−1^, characteristic of amide I and amide II, confirming the presence of proteins inherent to erythrocytes. The signal around 1450 cm^−1^ is associated with C–H stretching, while the band near 1080 cm^−1^ corresponds to C–O and C–O–C bonds, typical of polysaccharides such as tara gum and maltodextrin. Finally, the band around 703 cm^−1^ is related to more complex organic structures. In the encapsulated erythrocytes, these bands are maintained, although their intensity changes, indicating the incorporation of the material into the matrix.

The results demonstrate the presence of both the erythrocytes and the encapsulating matrix, indicating adequate incorporation of the core into the system. The preservation of the amide I and II bands suggests that the protein structure is maintained after encapsulation. Likewise, the characteristic signals of polysaccharides confirm the presence of tara gum and maltodextrin. Taken together, the changes in band intensity and their overlap indicate interactions between the matrix and the core, primarily via hydrogen bonds, which supports the success of the encapsulation process [22,27,28].

Figure 2b shows the FTIR spectra corresponding to the F1, F2, and F3 formulations of gummy candies incorporating encapsulated erythrocytes. In all formulations, a broad band is observed around 3388 cm^−1^, attributed to O–H groups in water and polysaccharides. The signals near 2934 cm^−1^ correspond to C–H stretching vibrations of organic compounds, such as sugars, gelatin, and encapsulated erythrocytes. In the region between 1651 and 1548 cm^−1^, bands related to protein structures are identified, attributable to both gelatin and erythrocytes. The signal around 1409 cm^−1^ is associated with C–H stretching, while the band at 1036 cm^−1^ corresponds to C–O and C–O–C bonds, characteristic of carbohydrates present in the formulation. Finally, the band near 712 cm^−1^ may be associated with out-of-plane C–H bending vibrations of organic compounds present in the gummy matrix.

The spectra exhibit very similar profiles across the formulations, suggesting that the incorporation of the encapsulated erythrocytes occurs primarily through a physical mechanism, without generating new chemical interactions. The presence of signals associated with hydroxyl groups, proteins, phenolic compounds, and sugars confirms that the matrix’s main components are preserved. Likewise, the similarity in band intensities indicates that the addition of encapsulated iron does not produce significant changes in the system’s functional groups. Taken together, these results demonstrate that the incorporation of encapsulated erythrocytes does not alter the matrix structure, maintaining its stability and functionality, thereby supporting its application in gummy-type products as a nutrient carrier [20,37,52,53].

### 3.4. Affective Sensory Evaluation of Fortified Gummy Candies

**Figure 3:**
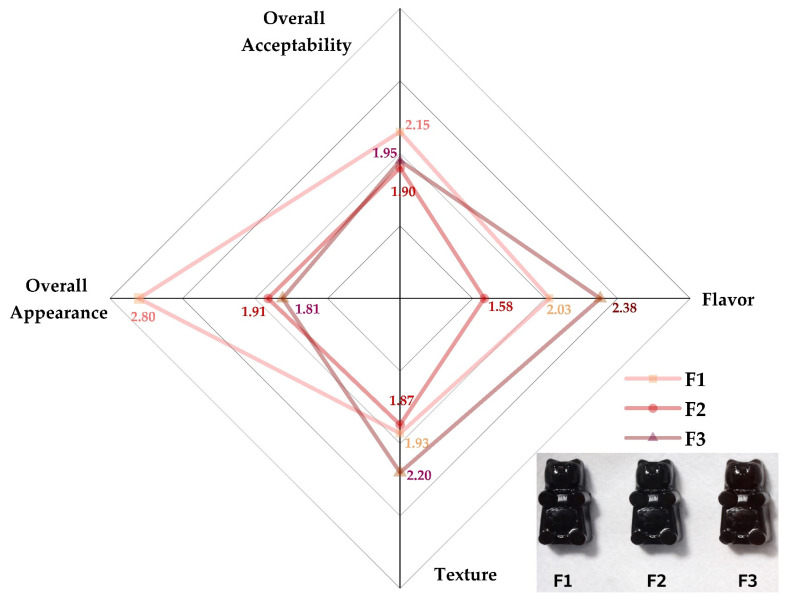
Sensory profile based on mean ranks of gummy candy formulations.

The affective sensory evaluation revealed differences among the formulations in overall appearance, flavor, texture, and overall acceptability (Figure 3), indicating distinct sensory profiles depending on composition. Statistically significant differences (*p* < 0.05) were observed only for the flavor attribute, according to the Friedman test (*p* = 0.002) and pairwise Wilcoxon comparisons, whereas overall appearance (*p* = 0.427), texture (*p* = 0.242), and overall acceptability (*p* = 0.115) showed no significant differences (*p* > 0.05).

Formulation F1 stood out in terms of overall appearance, which may be related to the higher visual intensity associated with its darker color. In contrast, formulation F3 performed better in terms of flavor and texture, indicating greater acceptance of attributes directly related to the product’s oral experience, which may be attributed to the proportion of the gelled matrix components. Meanwhile, formulation F2 showed the lowest overall sensory performance, failing to stand out in any of the evaluated attributes. Regarding overall acceptability, the three formulations showed similar values, indicating that, despite differences in individual attributes, all samples were accepted by the panelists; however, a non-significant tendency toward higher acceptability for F3 was observed, consistent with its better performance in taste and texture, attributes that typically carry greater weight in the consumer’s overall perception. This pattern is consistent with previous reports indicating that encapsulation can improve sensory properties by masking undesirable flavors and enhancing attributes such as taste and texture, thereby increasing product acceptability [11,54].

From a technological perspective, encapsulation preserved the system’s sensory quality by stabilizing the compounds responsible for the organoleptic profile, which is key to consumer acceptance and its potential commercial application [55,56,57]. Furthermore, this strategy facilitates the incorporation of functional ingredients, such as iron-rich erythrocytes, into gelled matrices without compromising sensory perception. Taken together, these results support the use of encapsulation in the development of fortified foods and its application for incorporating other bioactive compounds [58,59,60].

**Figure 4:**
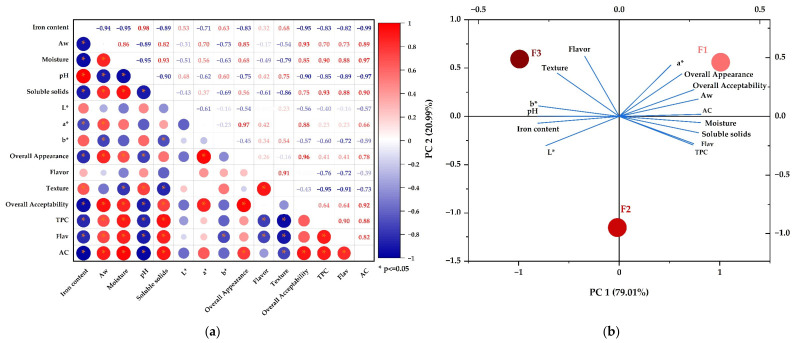
Heatmap (**a**) and principal component analysis (**b**).

The color map based on Pearson’s correlation analysis (Figure 4a) revealed multiple significant relationships (*p* ≤ 0.05) among the properties of the gummy candies. Iron content was negatively correlated with water activity, moisture content, total phenolic compounds, flavonoids, and antioxidant capacity, and positively correlated with pH. Water activity showed a direct relationship with moisture content and an inverse relationship with soluble solids. Similarly, moisture content was positively correlated with soluble solids and negatively correlated with pH, while pH also showed an inverse relationship with soluble solids. Regarding color parameters, a* was negatively correlated with iron content and positively correlated with water activity, while b* showed the opposite behavior. These results indicate that an increase in iron content, associated with a higher proportion of encapsulated erythrocytes, alters the system’s physicochemical behavior, reducing water availability and favoring higher pH values. Likewise, variations in the color parameters are influenced by changes in the composition and distribution of the matrix components, reflecting the effects of encapsulation and formulation on the product’s properties [20,23].

The principal component analysis (PCA) in Figure 4b enabled multivariate integration of the evaluated physicochemical and sensory variables [20,61,62]. The first two principal components accounted for the total variability of the system, with PC1 contributing 79.01% and PC2 20.99%. PC1 was primarily associated with variables related to water status and overall sensory perception, including water activity, moisture, soluble solids, overall acceptability, and general appearance, along with total phenolic compounds, flavonoids, and antioxidant capacity, in contrast to variables such as iron content and pH, which were located in the opposite direction. PC2, on the other hand, was dominated mainly by sensory attributes associated with the oral experience, such as taste and texture. Formulation F3 was associated with these sensory attributes, indicating better taste and texture performance than the other formulations.

Daily iron requirements vary with age, sex, and physiological status and are significantly lower than the therapeutic doses used to treat anemia; in children, these doses can range from approximately 3 to 6 mg/kg/day [63]. In this context, it has been reported that daily administration of a gummy containing heme iron (equivalent to 5 mg of elemental iron) for 21 days resulted in a significant increase in hemoglobin levels in children, with 93.9% of participants no longer having anemia at the end of treatment [64]. Based on the results obtained in this study, it is estimated that consuming approximately 5 to 6 gummy candies per day could contribute to meeting the daily iron intake for children, depending on their body weight and specific requirements; however, this estimate should be interpreted with caution, as factors such as iron bioaccessibility and potential bioavailability, the food matrix, and individual needs can significantly influence its absorption.

The absence of controls without iron, with non-encapsulated iron, and with conventional salts makes it difficult to attribute the observed changes solely to encapsulation or to the iron source. However, in preliminary tests, ferrous sulfate produced a pronounced metallic taste, so it was ruled out to prioritize sensory acceptability. This behavior has been described for ferrous salts due to their association with metallic notes, color changes, and oxidative interactions in food matrices [65,66]. In this context, microencapsulation was used as a strategy to improve iron’s compatibility with the gelled matrix and reduce undesirable sensory effects [6,17]. Therefore, the results should be interpreted as a comparison among the formulations developed, and future studies should include specific controls.

Although *Cavia porcellus* was used as a local source of heme iron due to its relevance as a food animal in the Peruvian Andean context, the proposed strategy could be adapted to other heme iron sources, such as bovine, porcine, ovine/caprine, or poultry blood. These alternatives should be obtained from authorized slaughterhouses and validated for safety, composition, sensory acceptability, and compatibility with the microencapsulation process.

## 4. Conclusions

This study demonstrated the feasibility of developing fortified gummy candies using a multicomponent iron system stabilized by microencapsulation and vacuum foam drying. The incorporation of the microencapsulated system increased the iron content from 0.21 to 0.89 mg Fe/g of gummy candy, while maintaining physicochemical properties within acceptable ranges and without compromising sensory acceptability.

The microencapsulated system showed adequate structural characteristics, while FTIR analysis suggested the preservation of functional groups associated with proteins and polysaccharide-based encapsulating materials, indicating compatibility among the matrix components. Differences in total phenolic compounds, flavonoids, and antioxidant capacity among formulations were mainly associated with the proportion of elderberry juice, confirming its contribution to the functional profile of the product. Likewise, the multivariate analysis showed consistent relationships among physicochemical, bioactive, and sensory variables, highlighting the influence of iron content and formulation composition on the behavior of the system.

Taken together, these findings suggest that vacuum foam drying is a promising technological strategy for incorporating iron into functional confectionery products. However, the results should be interpreted within the scope of a proof-of-concept study, since iron bioaccessibility, bioavailability, microbiological stability, and shelf-life were not evaluated. Therefore, future studies should address these aspects, optimize the formulation through conventional mixture designs, and further assess regulatory feasibility and consumer acceptance before practical application.

## Figures and Tables

**Table 1:** Formulation composition (%) of gummy candies.

Ingredients	Formulation		
	F1	F2	F3
Encapsulated erythrocytes (%)	10	14	18
Feranin^®^ (%)	1	2	3
Elderberry juice (%)	30	25	20
Water (%)	20	20	20
Neutral gelatin (%)	8	8	8
Sucrose (%)	10.5	10.5	10.5
Glucose (%)	14	14	14
Isomalt (%)	5	5	5
Sodium benzoate (%)	1	1	1
Glycerin (%)	0.5	0.5	0.5
Total (%)	100	100	100

**Table 2:** Physicochemical properties of gummy candies.

Properties	F1			F2			F3		
	$\bar{x}$	±	SD	$\bar{x}$	±	SD	$\bar{x}$	±	SD
Iron content (mg Fe/g of gummy candy)	0.21 ^a^	±	0.01	0.62 ^b^	±	0.01	0.89 ^c^	±	0.01
Water activity	0.88 ^a^	±	0.01	0.85 ^b^	±	0.01	0.84 ^b^	±	0.01
Moisture (%)	40.22 ^a^	±	0.56	37.44 ^b^	±	0.67	33.79 ^c^	±	0.97
pH	4.64 ^a^	±	0.16	5.41 ^b^	±	0.11	6.15 ^c^	±	0.10
Soluble solids (°Brix)	46.67 ^a^	±	0.58	45.00 ^a^	±	1.00	41.00 ^b^	±	1.00
L*	21.12 ^a^	±	0.36	21.45 ^a^	±	0.25	21.47 ^a^	±	0.24
a*	2.44 ^a^	±	0.17	1.29 ^b^	±	0.10	1.72 ^c^	±	0.02
b*	0.87 ^a^	±	0.09	0.90 ^a^	±	0.02	0.95 ^a^	±	0.01
Total phenolic compounds (mg GAE/g)	1.14 ^a^	±	0.04	1.11 ^a^	±	0.05	0.80 ^b^	±	0.02
Total flavonoids (mg QE/g)	0.27 ^a^	±	0.03	0.25 ^a^	±	0.03	0.13 ^b^	±	0.02
Antioxidant capacity (µmol ET/g)	3.75 ^a^	±	0.02	2.55 ^b^	±	0.01	1.53 ^c^	±	0.04

F1, F2, and F3 correspond to the gummy candy formulations. Results are expressed as mean ($\bar{x}$) ± standard deviation (SD) of three replicates (*n* = 3). Different letters within the same row indicate statistically significant differences (*p* < 0.05) according to one-way ANOVA and Tukey’s test.

## Data Availability

Data are available within the article.
